# Genome skimming approach reveals the gene arrangements in the chloroplast genomes of the highly endangered *Crocus* L. species: *Crocus istanbulensis* (B.Mathew) Rukšāns

**DOI:** 10.1371/journal.pone.0269747

**Published:** 2022-06-15

**Authors:** Selahattin Baris Cay, Yusuf Ulas Cinar, Selim Can Kuralay, Behcet Inal, Gokmen Zararsiz, Almila Ciftci, Rachel Mollman, Onur Obut, Vahap Eldem, Yakup Bakir, Osman Erol

**Affiliations:** 1 Department of Biology, Faculty of Sciences, Istanbul University, Istanbul, Turkey; 2 Department of Agricultural Biotechnology, Faculty of Agriculture, University of Siirt, Siirt, Turkey; 3 Department of Biostatistics, Erciyes University, Kayseri, Turkey; 4 Drug Application and Research Center (ERFARMA), Erciyes University, Kayseri, Turkey; 5 Department of Plant Bioactive Metabolites, ACTV Biotechnology, Inc., Istanbul, Turkey; Nigde Omer Halisdemir University, TURKEY

## Abstract

*Crocus istanbulensis* (B.Mathew) Rukšāns is one of the most endangered *Crocus* species in the world and has an extremely limited distribution range in Istanbul. Our recent field work indicates that no more than one hundred individuals remain in the wild. In the present study, we used genome skimming to determine the complete chloroplast (cp) genome sequences of six *C*. *istanbulensis* individuals collected from the *locus classicus*. The cp genome of *C*. *istanbulensis* has 151,199 base pairs (bp), with a large single-copy (LSC) (81,197 bp), small single copy (SSC) (17,524 bp) and two inverted repeat (IR) regions of 26,236 bp each. The cp genome contains 132 genes, of which 86 are protein-coding (PCGs), 8 are rRNA and 38 are tRNA genes. Most of the repeats are found in intergenic spacers of *Crocus* species. Mononucleotide repeats were most abundant, accounting for over 80% of total repeats. The cp genome contained four palindrome repeats and one forward repeat. Comparative analyses among other Iridaceae species identified one inversion in the terminal positions of LSC region and three different gene (*psbA*, *rps3* and *rpl22*) arrangements in *C*. *istanbulensis* that were not reported previously. To measure selective pressure in the exons of chloroplast coding sequences, we performed a sequence analysis of plastome-encoded genes. A total of seven genes (*accD*, *rpoC2*, *psbK*, *rps12*, *ccsA*, *clpP* and *ycf2*) were detected under positive selection in the cp genome. Alignment-free sequence comparison showed an extremely low sequence diversity across naturally occurring *C*. *istanbulensis* specimens. All six sequenced individuals shared the same cp haplotype. In summary, this study will aid further research on the molecular evolution and development of *ex situ* conservation strategies of *C*. *istanbulensis*.

## Introduction

*Crocus* is one of the largest genera of the family Iridaceae and consists of more than 200 species occurring from Western Europe and Northwestern Africa to Western China with the largest diversity in the Balkan Peninsula and Turkey [1, 2]. At present, the genus is represented in Turkey by 134 species, of which 117 are endemic, making it a biodiversity hotspot important for the conservation of *Crocus* species [1–4]. Some species of *Crocus* are economically important and have been used in the production of dye and perfume as well as in medicine. Despite their ecological and economic significance, most *Crocus* taxa are highly endangered because of anthropogenic activities such as mining, road construction, overgrazing, hydroelectric power stations, wind power stations and city expansion. The genus is characterized by slender grass-like leaves; white, yellow, blue, lilac or purple flowers; and corms with tunics. Since many of the diagnostic characters of this genus are relatively difficult to detect (such as characteristics of the underground corm and tunic, and the color and surface features of rarely collected seeds), integrative approaches including morphological and genetic analysis are now the preferred method for elucidating taxonomic ambiguities and phylogenetic questions [5].

*Crocus istanbulensis* (B.Mathew) Rukšāns was described by Mathew [1] as a subspecies of its relative *C*. *olivieri* J.Gay. Rukšāns [2] raised this taxon to the species level based on results by Erol & Küçüker [6]. *C*. *istanbulensis* is, one of the most endangered *Crocus* species in the world, having not been observed anywhere except in Istanbul. Its habitat is surrounded by highways, new human settlements and other anthropogenic activities resulting in soil alternation and destabilization. In particular, controversial forestation activities are a major factor in preventing the continued reproduction of *C*. *istanbulensis* because they destroy the soil and maquis vegetation of its habitat. During our last field trip to the *locus classicus* in winter of 2019, we found a total of only 25 individuals and it is estimated that no more than 100 individuals remain in the wild. The need to protect this plant is urgent and *in situ* and *ex situ* studies should start simultaneously to this end. To our knowledge, no genetic characterization studies have previously been carried out on *C*. *istanbulensis* and filling this knowledge gap was the primary motivation for this study. Analysing chloroplast genomes serves as a good starting point for the genetic characterization of this highly endangered species, as chloroplast genome sequences have been used extensively in plant molecular phylogenetics, population genetics and conservation genetics studies due to their slower rate of evolution compared with nuclear genomes, maternal inheritance and lower rate of recombination [7, 8]. Therefore, whole chloroplast genome sequences can provide a wealth of genetic information and are useful molecular markers for efficient conservation and management strategies [9–11]. Typically, the chloroplast genome maintains a conserved circular and quadripartite structure, with a pair of inverted repeat regions that are located between large single copy (LSC) and small single copy (SSC) regions, harbouring about 110–130 genes, with about 80 protein-coding genes, 4 rRNAs and 30 tRNAs.

Genome skimming is a rapid and cost effective strategy for recovering plastid and mitochondrial genomes using next generation sequencing technology [12, 13]. In this study, we sequenced the chloroplast genome sequences of six specimens of *C*. *istanbulensis* using DNA nanoball and combinatorial probe anchor synthesis on the BGI-Seq 500 platform. Our main objectives were to: (i) obtain information regarding the sequence and structural characterization of *C*. *istanbulensis* cpDNA, (ii) test whether complete chloroplast genomes in *C*. *istanbulensis* demonstrates structural rearrangements compared with other Iridaceae taxa and (iii) detect whether the genes underwent positive selection.

## Methods

### Plant sampling and total DNA extraction

Specimens were collected in January 2019 from Taşdelen state forest in the Çekmeköy district in Istanbul, Turkey. Permission for collecting specimens was granted by Republic of Turkey Ministry of Agriculture and Forestry (No:53231444–100.05–4722). Due to the extremely low number of individual and limited distribution area of about 4000 m^2^, only leaves of eight plant specimens were collected for total DNA isolation, the corms were not dug up or disturbed. Since the meristematic elongation zone of *Crocus* leaves is located at the leaf base, the leaves continued to grow and develop afterwards. Sampling was done in a way that would cause the least possible damage to the plant. The leaves were immediately frozen in liquid nitrogen and stored at −80°C until DNA extraction. Approximately 750 mg of freshly frozen leaves were used for DNA extraction according to Healey [14]. The DNA concentration of each sample was measured using Qubit dsDNA HS Assay Kit (Life Technologies). DNA purity was assessed by measuring A260/280 absorbance ratio using a Nanodrop ND-2000c spectrophotometer (Nanodrop Technologies) and agarose gel electrophoresis to ensure high-molecular-weight DNA integrity. Only six DNA samples that had a A260/280 value between 1.7 and 1.9, and a concentration of >200 ng/μl (in total volume ~40 μl) were selected for library preparation and sequencing.

### DNA sequencing

Prior to library constructions, six qualified DNA samples were fragmented into 150–250 bp fragments using Covaris technology, then fragment size distributions were checked using the QIAxcel Advanced System (Qiagen) and quantified using the Qubit dsDNA HS Assay Kit (Life Technologies). End-repair of DNA fragments, addition of an adenine residue to the 3′ fragment ends, adaptor ligation, and rolling circle amplification (RCA) were performed according to MGIEasy FS DNA Library Prep Set. Each DNA nanoballs (DNBs) were loaded onto a sequencing flow cell and then processed for 101 bp paired-end sequencing on the BGISEQ-500 platform. The raw image files obtained from the sequencing were processed using BGISEQ-500 basecalling software and the raw sequence data were saved in ".fastq" format. The raw fastq files were deposited in the Sequence Read Archives (SRA) of the National Center Biotechnology Information (NCBI) under Bioproject number PRJNA599306.

### Genome assembly and annotation

Before *de novo* chloroplast genome assembly, raw sequencing reads were subjected to pre-processing and quality control using AfterQC v0.9.7 [15] by the following steps: removing adapter sequences, discarding the low quality reads (Phred quality score less than 20, Q ≥ 20) and ambiguous nucleotides (‘N’ at the end of reads) and discarding short length reads (<50 bp). High-quality reads were used for *de novo* chloroplast assembly using SPAdes v3.13.0 [16] and visualized using Bandage v0.8.1 [17], integrated into GetOrganelle pipeline (https://github.com/Kinggerm/GetOrganelle) [18]. *C*. *cartwrightianus* (NC_041459) and *C*. *sativus* (NC_041460) species were included as reference species. Chloroplast genome annotation (protein coding, rRNA, and tRNA genes prediction) was performed by a combination of CPGAVAS [19] and GeSeq [20], and a circular map of the genome was generated with OGDRAW v1.3.1 [21]. The length and locations of forward, reverse, palindromic and complementary repeats in the *C*. *istanbulensis* chloroplast genome were determined by REPuter web-service (https://bibiserv.cebitec.uni-bielefeld.de/reputer/) with a minimum repeat size 30 bp and a sequence identity of 90% (Hamming distance = 3). The identification and localization of simple sequence repeats (SSRs) were carried out using MISA perl script (http://pgrc.ipk-gatersleben.de/misa/misa.html) with default parameters. The minimum numbers for the microsatellite motifs were 10, 5, 4, 3, 3 and 3 for mono-, di-, tri-, tetra-, penta, and hexanucleotide repeats, respectively.

### Comparative chloroplast genome analysis in Iridaceae

To infer evolutionary events such as sequence divergence, gene order rearrangements, the expansion and contraction of the inverted repeats in Iridaceae, we used the online webtool Irscope [22] to compare the complete cpDNA of *C*. *istanbulensis* with *C*. *sativus* L., *C*. *cartwrightianus* Herb., *Iris missouriensis* Nutt., *Iris sanguinea* Donn ex Hornem., *Iris gatesii* Foster and *Geosiris australiensis* B.Gray & Y.W.Low. Using the Irscope tool, we found and visualized the structural organization of junction sites connecting two inverted repeats (IRs) to long single-copy (LSC) and short single-copy (SSC) regions within Iridaceae [22]. We used the geneCo [23] software for the construction of a genome map and genome map comparison between *Crocus* species. To measure genetic distance and divergence between six *C*. *istanbulensis* individuals and other Iridaceae species, we applied an alignment-free, kmer-based approach using the accurate genomic distance estimation feature of Skmer v3.2.1 [24].

### Positive selection analysis of PCGs in Iridaceae

For the accurate detection of site-specific positive selection in the protein-coding sequences of Iridaceae, a Nextflow pipeline, which is a scalable and reproducible scientific workflow designed for positive selection analysis, called “PoSeiDon” [25] was employed using default parameters. Briefly, the orthologous protein-coding sequences of seven Iridaceae species were manually extracted from GenBank files (“.gbk”) and validated using SwiftOrtho [26]. Following in-frame alignment, indel correction and the calculation of phylogenetic tree, the best-fitting nucleotide substitution model was selected using MODELTEST. Then, positively selected sites (*ω*>1) under varying models M1a vs. M2a, M7 vs. M8 within the PAML suite (v4.9) and M8a vs. M8 by Swanson et al. (2003) [27] were tested using three independent codon models F1X4, F3X4, F6. After this calculation, we used a Bayes empirical Bayes (BEB) approach [28] to calculate posterior probability (PP) of a codon coming from a site class of *ω*>1. Genes were considered to be positively selected if positively selected sites (*ω* >1) were assigned a PP > 0.95.

## Results and discussion

### Chloroplast genome assembly and annotation

**Fig 1:**
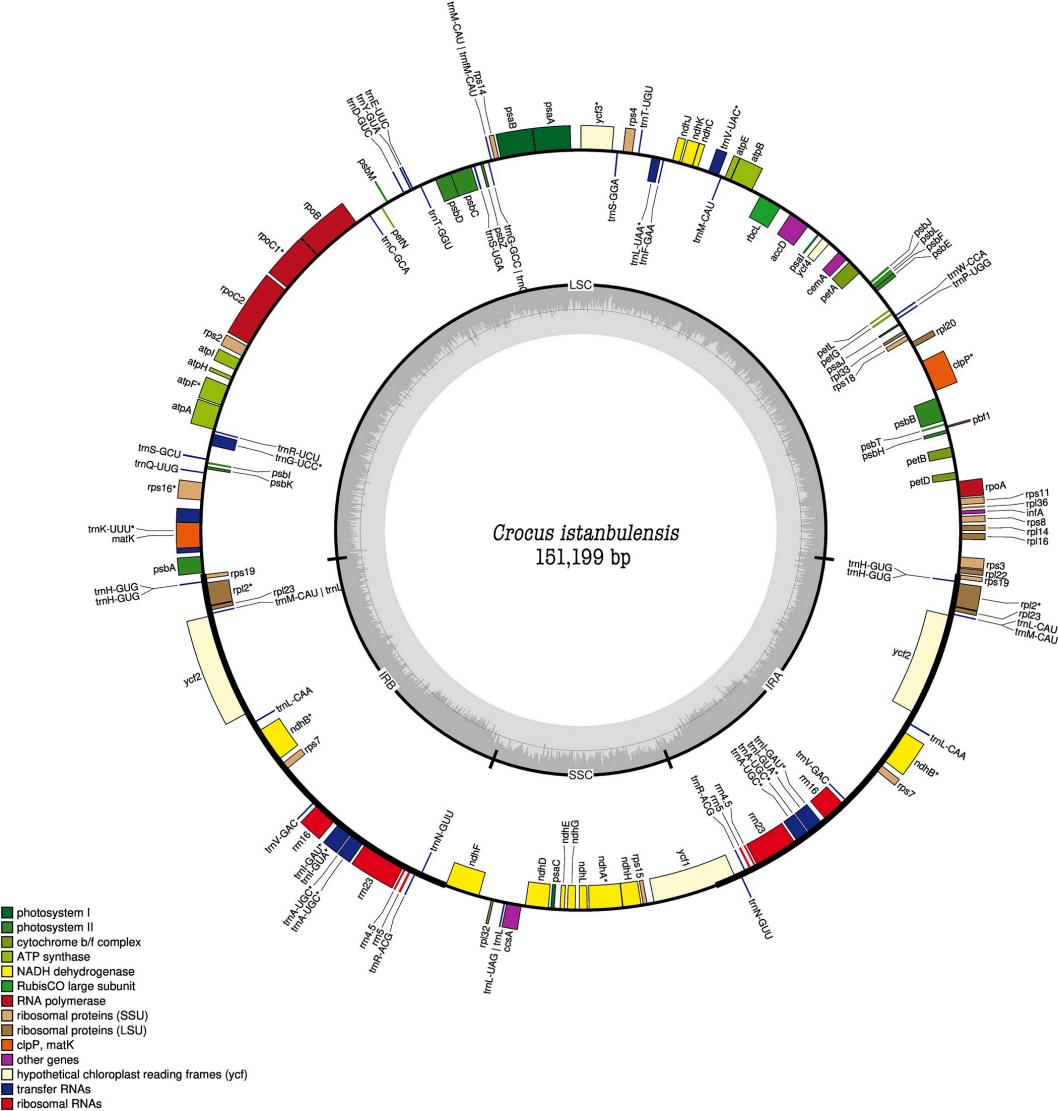
Circular visualization of cp genome annotation for *C*. *istanbulensis*. Genes belonging to different functions categories were shown in different colors. Genes drawn inside the circle are transcribed clockwise, and those outside are transcribed counter clockwise. GC content ratio is shown in the middle circle.

After trimming of adaptor sequences and low-quality sequences, a total of 114.1 million clean reads comprising 11.41 gigabases (Gb) were generated from *C*. *istanbulensis* specimens. On average 1.90 Gb were generated per individual, with a mean sequencing depth of 532X (S1 Table) and the sequence of the chloroplast genome was registered into GenBank with the accession number MN254968. The percentage of reads covering the chloroplast genome was between 8.56% (~73 million bases) and 8.44% (~94 million bases), the average being 8.47% (~81 million bases) (S1 Table). The entire chloroplast genome of *C*. *istanbulensis* consisted of 151,199 bp nucleotides, divided into four regions, which included a LSC region of 81,197 bp, a SSC region of 17,524 bp, separated by two inverted repeats (IR) regions of 26,239 bp each. These lengths were found be consistent with previous studies [29]. Previous cp genome studies suggest that angiosperm cp genomes are highly conserved, typically about 115–165 kb in size and a quadripartite structure with two IR regions (IRa and IRb), a LSC region and a SSC region [30]. The overall GC content of the *C*. *istanbulensis* cp genome was 37.6%. Among the LSC, SSC and inverted repeat regions, the highest GC content was found in the IR regions (42.75%), and GC contents of the LSC and SSC regions were 35.69%, and 30.97%, respectively. The IR region had an overall higher GC content due to the presence of more of rRNA and tRNA genes, which have high GC content (Table 1). This result was compatible with previous findings on the complete cpDNA of *Crocus* and *Iris* species [31–33]. Through gene annotation, we found that the cp genomes encode 132 genes, including 86 protein-coding genes (PCGs), 8 rRNA genes and 38 tRNA genes (Fig 1, Table 1).

**Table 1:** Chloroplast genomes features of seven taxa from the Iridaceae.

	*Crocus istanbulensis*	*Crocus cartwrightianus*	*Crocus sativus*	*Iris sanguinea*	*Iris gatesii*	*Iris missouriensis*	*Geosiris australiensis*
Genome Size (bp)	151,199	150,819	150,820	152,408	153,441	153,084	119,004
**LSC (bp)**	81,197	81,309	81,310	82,340	82,659	82,484	45,795
**IR (bp)**	26,239	26,057	26,056	26,026	26,221	26,168	36,347
**SSC (bp)**	17,524	17,396	17,396	18,016	18,376	18,264	515
**Number of Genes**	132	132	132	133	132	133	111
**Number of PCGs**	86	86	86	87	86	86	39
**Number of tRNAs**	38	38	38	38	38	38	37
**Number of rRNAs**	8	8	8	8	8	8	8
**Genome GC%**	37.6	37.5	37.5	38.5	37.9	37.9	38.5
**LSC GC%**	35.69	35.57	35.57	36.23	36.01	36.07	35.79
**IR GC%**	42.75	42.79	42.79	43.07	43.07	43.08	40.33
**SSC GC%**	30.97	30.76	30.76	31.83	31.55	31.52	31.59
**Accession No.**	MN254968	MH542231	MH542233	KT626943	KM014691	MH251636	MH142524

The LSC region includes 62 protein-coding and 21 tRNA genes, while SSC includes 12 protein-coding and 1 tRNA genes. The IRa and IRb regions include 6 protein-coding genes 8 tRNA genes, and 4 rRNA genes (S2 Table). In other words, 6 protein-coding genes, 8 tRNA genes, and 4 rRNAs were duplicated in the IR regions. As expected, cp genes are functionally classified into four categories (Table 2), of which the photosynthetic pathway contains the most PCGs. All but 9 of the PCGs did not contain introns, and of these 5 (*atpF*, *ndhA*, *rps16*, *rpoC1* and *clpP*) contain 1 intron, while 4 (*rps12*, *ndhB*, *ycf3* and *rpl2*) contain 2 introns (Table 2). As in a previous study, 3 genes (*rps12*, *clpP*, and *ycf3*) were found to possess 2 introns [29]. Moreover, *rps12* was found to be a trans-spliced gene [34]. The longest intron with a length of 2,639 bp was *trnK-UUU*, which is found in the *matK* gene (Fig 1). *matK* coding sequence (CDS) and many other regions were tested for species identification and phylogeny reconstruction [35, 36]. The non-coding sequence *trnH* (GUG)-*psbA* was found to be variable and thus useful for phylogeny and it has better resolution potential than *matK* and *rbcL* [36]. Such variable regions have the potential for *Crocus* species delimitation or phylogeny studies in future work.

**Table 2:** The functional classification of cp genes annotated in the cp genome of *C*. *istanbulensis*.

Category	Gene group	Gene Name
**Genes for photosynthesis**	Subunits of Photosystem I	*psaA*, *psaB*, *psaC*, *psaJ*, *psaL*
	Subunits of Photosystem II	*psbA*, *psbC*, *psbD*, *psbE*, *psbF*, *psbH*, *psbI*, *psbJ*, *psbK*, *psbL*, *psbM*, *psbT*, *psbZ*
	Large subunit of rubisco	*rbcL*
	Subunits of ATP synthase	*atpA*, *atpB*, *atpE*, atpF*, *atpH*, *atpL*
	Subunits of cytochrome	*petA*, *petB*, *petD*, *petG*, *petL*, *petN*
	Subunits of NADH dehydrogenase	ndhA*, *ndhB*^†^ (x2), *ndhC*, *ndhD*, *ndhE*, *ndhF*, *ndhG*, *ndhH*, *ndhL*, *ndhJ*, *ndhK*
**Self-replication**	Small subunit of ribosome	*rps2*, *rps3*, *rps4*, *rps7* (x2), *rps8*, *rps11*, *rps12*^†^, *rps14*, *rps15*, rps16*, *rps18*, *rps19* (x2)
	Large subunit of ribosome	*rpl2*^†^ (x2), *rpl14*, *rpl16*, *rpl20*, *rpl22*, *rpl23* (x2), *rpl32*, *rpl33*, *rpl36*
	Transfer RNA genes	*trnP-UGG*, *trnW-CCA*, *trnM-CAU*, *trnI-GAU*, *trnF-GAA*, trnL-UAA*, *trnT-UGU*, *trnS-GGA*, *trnM-CAU*, *trnG-UCC*, *trnS-UGA*, *trnT-GGU*, *trnE-UUC*, *trnY-GUA*, *trnD-GUC*, *trnC-GCA*, *trnR-UCU*, *trnS-CGA*, *trnS-GCU*, *trnQ-UUG*, trnK-UUU*, *trnH-GUG*, *trnM-CAU*, *trnL-CAA* (2x), *trnE-UUC*, *trnA-UGC* (x2)*, *trnR-ACG*, *trnN-GUU*, *trnL-UAG*, *trnN-GUU*, *trnR-ACG*, *trnE-UUC*, trnV-GAC* (2x), *trnL-CAA*, *trnM-CAU*, *trnH-GUG*
	DNA-dependent RNA polymerase	*rpoA* (x2), *rpoB* (x2), rpoC1* (x2), *rpoC2*
**Other genes**	Translational initiation factor	*infA*
	Protease	*clpP* *
	Maturase	*matK*
	Envelop membrane protein	*cemA*
	Subunit of acetyl-CoA-carboxylase	*accD*
**Unknown**	Conserved hypothetical chloroplast reading frames	*ycf1*, *ycf2* (x2), *ycf3*, *ycf4*

* indicates gene containing a single intron, (2X) refers genes that are located in the IRs and hence are duplicated.

### Junction characteristics, IR expansion, and contraction

**Fig 2:**
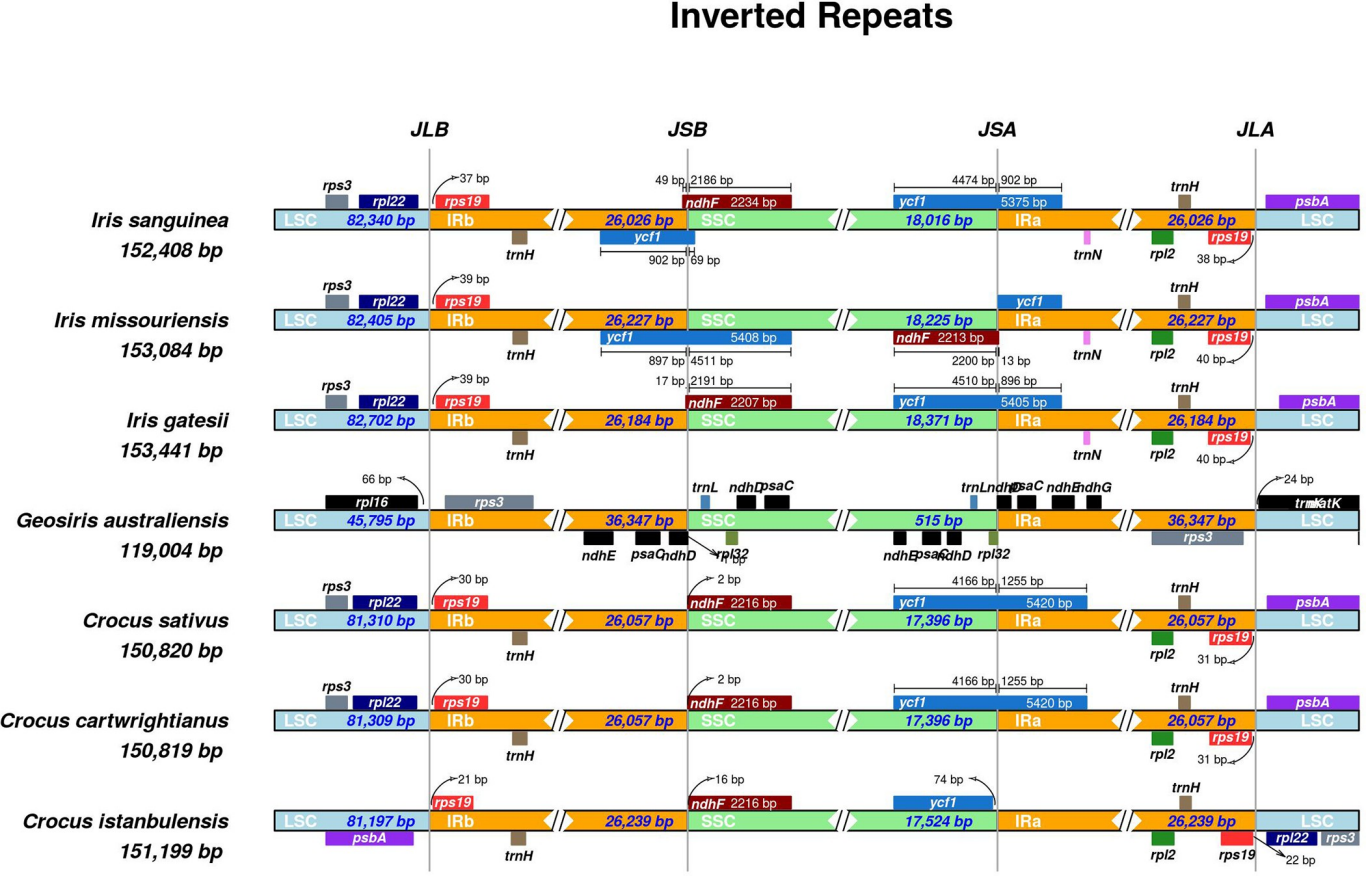
Comparison of the LSC, IR and SSC junction positions among seven-chloroplast genome of Iridaceae. JLB represents the of LSC/IRb junction, JSB represents the IRb/ SSC junction, JSA represents the SSC/IRa junction, and JLA represents the IRa/LSC junction. The thin lines represent the connection points of each area, and the number of base pairs (bp) show the distance from the boundary site to the end of the gene (in colored box).

Although the chloroplast sequences of flowering plants generally conserve a typical quadripartite structure, rearrangements or contractions/expansions of inverted repeats and single copy regions can lead to changes in genome size and allow certain genes to enter the inverted region (IR) or single copy region (SCR). Accordingly, the contraction and expansion of the two IR regions can be thought of as an indicator of chloroplast genome evolution, especially between closely related genera [37, 38]. We compared the inverted repeats and single copy regions boundaries of the seven Iridaceae chloroplast genomes (*C*. *istanbulensis*, *C*. *cartwrightianus*, *C*. *sativus*, *I*. *missouriensis*, *I*. *sanguinea*, *I*. *gatesii* and *G*. *australiensis*) (Fig 2).

**Fig 3:**
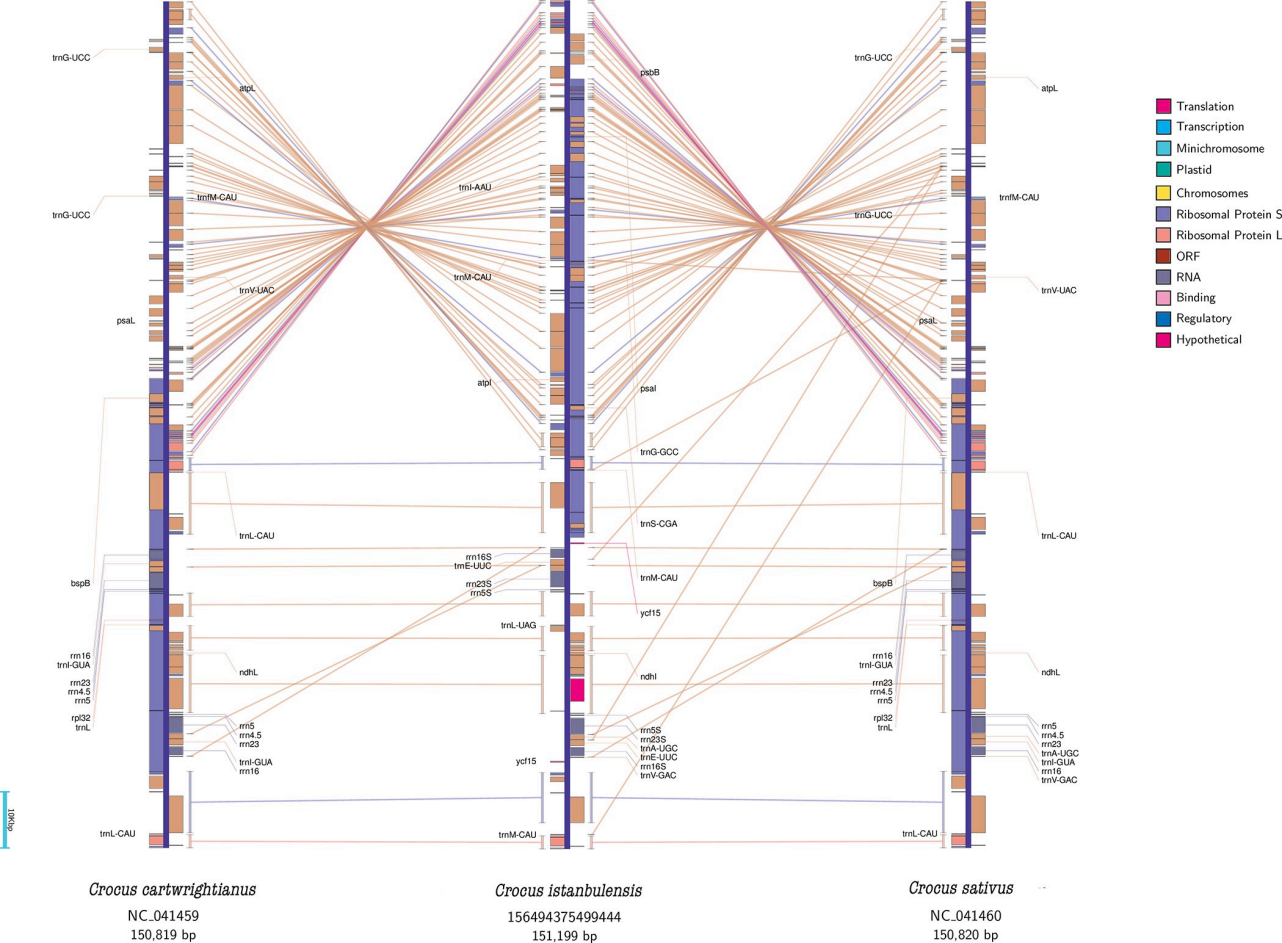
Comparative genome map of *C*. *cartwrightianus*, *C*. *istanbulensis* and *C*. *sativus*. Lines among cp genomes represent matched genes. Genes were colored based on their structural and functional classes, the inverted region of 26,239 bp in length.

Although the IR boundary regions varied slightly, they all generally fit the quadripartite structure pattern. Moreover, we observed no significant change in contraction and expansion of inverted repeats (IRs), except for in *G*. *australiensis*, whose LSC and SSC regions were contracted and IRb/a regions were expanded nearly 1.5 fold. In general, most size changes in the cp genomes of angiosperms can be explained by rare deletions and duplications that result in massive changes in the size of the IR region [39]. A notable difference was found in *psbA*, *rps3* and *rpl22* gene arrangements among *Crocus* species, indicating an inversion or reversal of gene order in LSC region terminal positions (Fig 2). To obtain more precise information about cp genome arrangements, a genome map comparison analysis was carried out with a genbank annotation file (“.gbk”) of *Crocus* species. Comparison analysis clearly indicates an inversion at the junction site of the LSC region (Fig 3).

Moreover, as can be seen in S1 Fig, *rps19* and *psbA* genes are located in the flanking region of the LSC/IRb junction and the *rpl22* gene is located in the LSC terminal region close to IRa in *C*. *istanbulensis*. In *C*. *cartwrightianus* and *C*. *sativus*, *rps19* and *psbA* are located in the flanking region of the LSC/IRa boundary and *rpl22* is located in the LSC terminal region close to IRb (S1 Fig). One other intriguing observation is that the *ycf1* gene (5420 bp) in *C*. *cartwrightianu*s and *C*. *sativus* is located within the SSC/IRa boundary and expanded upstream and downstream by 4166 bp and 1255 bp, respectively. However, the *ycf1* gene of *C*. *istanbulensis* is located within the SSC region and separated from the SSC border by 74 bp (Fig 2). Expansion and contraction of IRs in the organelle genome (cpDNA) of most angiosperms have been proposed as evolutionary dynamics parameters/markers for illuminating relationships between some plant taxa [40, 41]. IRs are also potential evidence of a duplication event prior to the separation of monocot lineages from basal angiosperms [42]. The absence of IRs in some plant groups, particularly legumes [43] and a decrease of up to 495 bp in *Pinus thunbergii* Parl. [44] suggest that these IRs are not required for chloroplast function. However, it is also thought that IRs are essential for the constant and stable nature of chloroplast genomes. Particularly, structural rearrangements such as inversions, IR expansions and gene duplication directly govern the structural organization and size of the chloroplast genome. Although the mechanisms leading to rearrangements in chloroplast genome are poorly known, intramolecular homologous recombination governed by the presence of repeat structures at the boundaries of the rearranged region reportedly plays a role in such structural changes [45, 46]. As indicated in Figs 2 and 3, the *C*. *istanbulensis* cp genome contains an inversion in the terminal position of the LSC region and a rearrangement of the *psbA*, *rpl22* and *rps3* gene order. It is noteworthy that this kind of arrangement has not previously been reported in Iridaceae cpDNAs. These results bring new insights into the evolution of the cp genome in *Crocus* genera, suggesting a need for further studies to understand how the ecological drivers, morphological traits and physiological functions of *C*. *istanbulensis* may relate to such rearrangements. Recent studies also showed that two chloroplast structural haplotypes (inverted and canonical haplotypes) can occur in most land plants. Long-read sequencing approaches such as PacBio or Oxford Nanopore may be helpful in determining the haplotype structure [47]. Although this study found only inverted haplotypes, third-generation sequencing may reveal the presence of a canonical haplotype in *C*. *istanbulensis*.

### Repetitive sequences analysis

**Fig 4:**
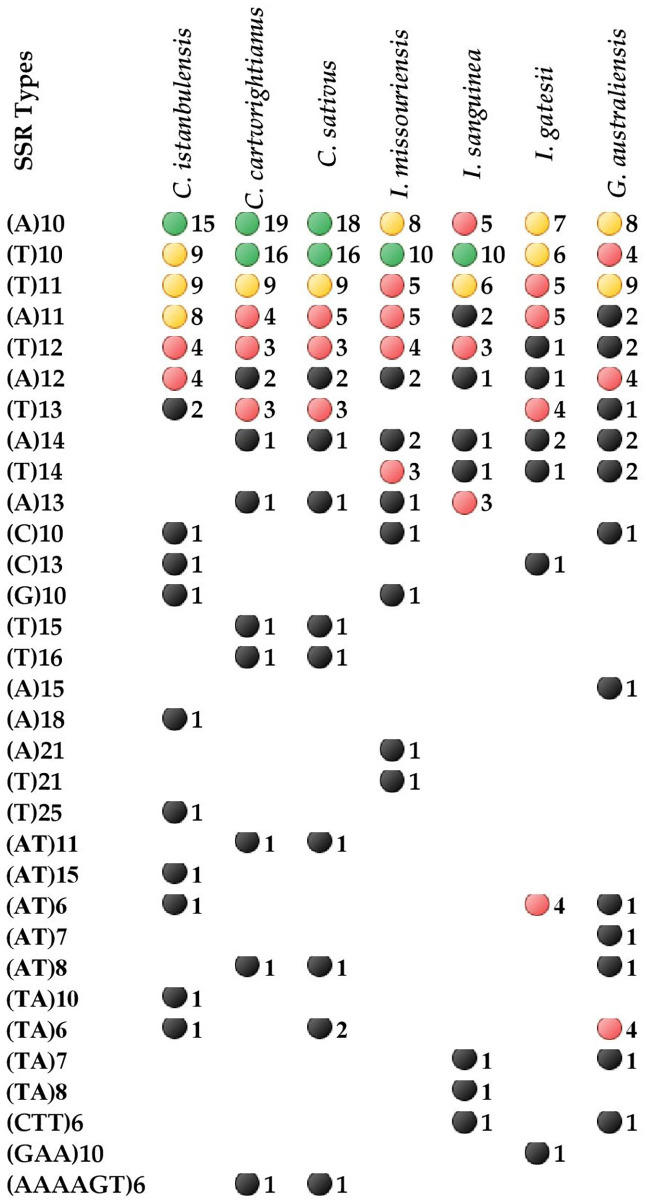
The distribution of SSR motifs in Iridaceae cp genome. Those marked with green, yellow, red and black circle indicate high, middle, low and lowest SSR numbers.

SSRs resulting from slipped strand mispairing during DNA replication are usually determined in organelle genomes and have been shown to have significant usage potential in plant population genetics and crop breeding studies [48]. In the current study, the online version of REPuter software was used to analyze forward, palindrome, reverse and complement repeat sequences of the Iridaceae cp genome, with a minimum repeat size of 30 bp and a sequence identity greater than 90%. An average of eight repeats with lengths of nearly 41 bp were observed in Iridaceae species. *C*. *istanbulensis* contained four palindrome repeats and one forward repeat (S3A Table). Overall, four repeats were 30–32 bp long, with one repeat 52 bp long. A previous study on two species from Lauraceae, *Machilus balansae* S.K.Lee & F.N.Wei and *M*. *yunnanensis* Lecomte, found a similar number of repeats varying from 39 to 41 bp (with lengths of 20 bp) [49]. *C*. *cartwrightianus* and *C*. *sativus* contained three forward repeats and three palindrome repeats. Two repeats were 30–40 bp long and, four repeats were 40–56 bp long (S3B and S3C Table). Other Iridaceae species (*I*. *missouriensis*, *I*. *gatesii* and *G*. *australiensis*) seem to have more repeat sequences in terms of both number and size, except for *I*. *sanguinea* (S3D–S3G Table). Many repeats shared the same locus in Iridaceae: *ycf1*, *ycf2*, *accD* and *petN*-*psbM*, *psaC*-*ndhE*, *ndhD*–*psaC*, *psbA*-*rps19*, *psbM*–*petN* and *rps16*-*trnQ-UUG* intergenic spacer (S3 Table). According to previous studies, cp-SSR regions show variable profiles generally without recombination, are uniparentally inherited and effectively haploid, and are used for genetic studies of plant populations [50, 51]. Most of the repeat profiles are found in the intergenic spacer of *Crocus* species in the current study. This situation corroborates previous plant genome studies [52, 53]. As for SSR number and motif distribution, SSRs occupied 0.49% and 0.26% of the total cp genome respectively, with an average of 0.39% (Table 3). Regardless of species, mononucleotide repeats were most abundant and accounted over 80% of total repeats, which contained mostly A/T mononucleotide motifs (Table 3, Fig 4).

**Table 3:** The number and distribution of SSR repeats in Iridaceae cp genome.

		*Crocus istanbulensis*	*Crocus cartwrightianus*	*Crocus sativus*	*Iris sanguinea*	*Iris gatesii*	*Iris missouriensis*	*Geosiris australiensis*
Total	**Number**	60	63	65	44	35	38	45
	**Total Size (nt)**	699	724	749	507	401	449	516
	**% of cpDNA**	0.46	0.48	0.49	0.33	0.26	0.29	0.43
Monomer	**Number**	56	60	60	44	32	33	36
	**Total Size (nt)**	625	650	651	507	353	371	407
	**% of repeats**	89.41	89.78	86.92	100.00	88.03	82.63	78.88
Dimer	**Number**	4	2	4	0	2	4	8
	**Total Size (nt)**	74	38	62	0	30	48	91
Trimer	**Number**	0	0	0	0	1	1	1
	**Total Size (nt)**	0	0	0	0	18	30	18
Hexamer	**Number**	0	1	1	0	0	0	0
	**Total Size (nt)**	0	36	36	0	0	0	0

Only a minor fraction consisted of dinucleotide, trinucleotide, and hexanucleotide repeat motifs. Among dinucleotides, the number of repeats ranged from two (*I*. *sanguinea*, *C*. *cartwrightianus*) to eight (*G*. *australiensis*). One trinucleotide repeat (CTT, GAA) was detected in *I*. *sanguinea*, *I*. *gatesii*, *G*. *australiensis*. Tetra-, and pentanucleotides were not found in any Iridaceae, but hexanucleotide repeats were only present in *C*. *cartwrightianus* and *C*. *sativus* cp genomes (Table 3, Fig 4).

### Identification of positive selection genetic signatures in cp coding genes of *C*. *istanbulensis*

To gain additional insight into potential changes in selection pressure in the exons of chloroplast coding sequences over the course of evolution of *C*. *istanbulensis*, we compared these genes across the six publicly available Iridaceae species. Here, we applied site‐specific models with three comparison models (M1a vs. M2a, M7 vs. M8, M8a vs. M8) likelihood ratio test (LRT) (threshold value *p* < 0.01) in PoSeiDon pipeline (PP > 0:95). Currently, the signature of selection pressure (or evolutionary rate *ω*) can be detected by comparing the rate of non-synonymous (dN) and synonymous substitutions (dS) in alignment of orthologous sequences. The ratio is often used to assess the strength and direction of natural selection acting on protein-coding genes throughout nuclear and organelle genome [54–56]. This approach is generally used to demonstrate whether there are any positive selection pressures in organelle-coding genes. However, this approach does not take possible recombination events into account [25]. Although it is commonly stated that recombination events do not occur in chloroplast genomes, accumulating evidence of recombination events shows that chloroplast genomes do have the potential to alter their genome structure via recombination [50, 57–59]. Therefore, we used PoSeiDon pipeline, a new approach that takes recombination events into account [25]. Among 86 protein-coding genes, our analysis found signatures of positive selection in seven genes *accD* (*PP* = > 0.99), *rpoC2* (*PP* = > 0.99), *psbK*, *rps12*, *ccsA*, *clpP* and *ycf2* (Table 4). Caseinolytic protease (CLP) and acetyl-coA carboxylase (ACCase) are two enzymes required for proper plastid function and fatty acid biosynthesis. The CLP complex and ACCase genes encode subunits of plastid-encoded *accD* and *clpP* genes, respectively [60–62]. Although *clpP* and *accD* are generally well conserved, recent findings indicate that the plastid-encoded version of these genes have elevated rates of sequence evolution in multiple independent lineages [54, 63, 64]. In this study, we found the signatures of intense positive selection acting on plastid-encoded *accD* and *clpP* genes, which have effects on leaf longevity and seed yield, and are essential for plant cell viability, respectively [54, 65]. Zeng et al. [66] attributed the positive selection in *clpP* genes to plant acclimation to different physiological conditions and reported that the high degree of positive selection observed in *clpP* may be important in adapting *Rehmannia* species to habitats with different light intensities. We also found positive selection on photosystem II (PSII) reaction center protein K (*psbK*) gene, which encodes one of the components of the core complex of PSII, which functions in both light-harvesting and inducing the oxidation of water to dioxygen [67, 68]. Because *psbK* is directly involved in PSII, the positive selection observed in the *psbK* gene of various plants such as *Echinacanthus* Nees. [69], *Robinia* L. [70], *Debregeasia* Gaudich [71], Monsteroideae (Araceae) [72] and *Garcinia paucinervis* Chun & F.C.How [73] are important for plant adaptation to harsh environmental conditions. A significant positive selection signature was also detected in *ccsA* gene, which encodes a component of cytochrome c synthase complex for cytochrome c biogenesis [74] and has been reported to play a role in the adaptation of species to environmental conditions [75–77]. Interestingly, we also identified 3 genes with positive selection sites (*rpoC2*, *ycf2* and *rps12*). The *rpoC2* gene encodes subunits of plastid-encoded plastid RNA polymerase, responsible for photosynthetic gene expression. In other words, it allows for transcription of photosynthesis-related genes in the chloroplast. These plastid-encoded genes are also considered relatively rapidly evolving regions [78]. The *ycf2* gene is one of the largest genes encoding for a putative membrane protein in the chloroplast. There is accumulating evidences suggesting that these two genes may have rapidly evolved in various plant cp genomes and enhance adaptation to diverse environments, possibly as a result of altered transcription [55, 76, 79–83]. Apparent positive selection signatures were found in seven genes (*accD*, *rpoC2*, *psbK*, *rps12*, *ccsA*, *clpP* and *ycf2*) in the *C*. *istanbulensis* chloroplast genome. Previous studies indicated that many of these putatively positively selected genes were associated with plastid function, fatty acid biosynthesis, leaf longevity, seed yield, cell viability, adaptation to challenging environmental conditions and photosynthesis. Although the function of the seven positively selected genes in *C*. *istanbulensis* remains unknown and requires further experimental validation, we speculate that they might be involved in biological processes including photosynthesis, environmental stress response, and plant development and growth.

**Table 4:** Results of the evolutionary analyses for positively selected sites for *accD*,.

Gene	Region		M7 vs M8 (χ^2^)	M7 vs M8 *p*-value	% sites with *ω >* 1	avg(*ω*)	M8 BEB (*PP* > 0:95 = > 0.99)
*accD*	**F61**	Full (aa 1–442)	26.89	< 0:001	1.12	34.13	R4; M34; L38; L55; A212; **N234**; Q392; **R438**; **K440**; **R441**; **N442**
	**F1X4**	Full (aa 1–442)	24.03	< 0:001	1.01	41.67	**R438**; **K440**; R441; **N442**
	**F3X4**	Full (aa 1–442)	21.47	< 0:001	3.14	11.79	**R438**; K440; **N442**
*rpoC2*	**F61**	Full (aa 1–1355)	6.46	0:04	12.65	2.24	I626; **Q925**; **E952**; **N1155**; S1355
	**F1X4**	Full (aa 1–1355)	2.77	0:25	NA	NA	NA
	**F3X4**	Full (aa 1–1355)	5.33	0:07	1.15	6.38	Q925; **E952**; N1155; S1355
*psbK*	**F61**	Full (aa 1–61)	6.0	0:05	23.95	6.08	S17
	**F1X4**	Full (aa 1–61)	8.19	0:017	4.71	31.82	S17; H20
	**F3X4**	Full (aa 1–61)	10.56	0:005	6.38	26.49	S17; H20
*rps12*	**F61**	Full (aa 1–116)	28.54	< 0:001	25.78	14.87	**M1**; **T5**; **R6**; Q7; N11; S15; P16; C21; G26; **T27**; C28
	**F1X4**	Full (aa 1–116)	33.88	< 0:001	25.57	16.27	**M1**; **T5**; **R6**; Q7; N11; V12; S15; **P16**; C21; **G26**; **T27**; C28; V31
	**F3X4**	Full (aa 1–116)	37.64	< 0:001	25.44	20.04	**M1**; T5; R6; Q7; N11; V12; S15; **P16**; C21; G26; T27; C28; V31
*ccsA*	**F61**	Full (aa 1–318)	5.23	0:073	1.99	6.96	A4; G92; A103
	**F1X4**	Full (aa 1–318)	9.13	0:01	1.68	10.5	A4; **G92**; A103
	**F3X4**	Full (aa 1–318)	8.44	0:015	1.86	9.71	A4; **G92**
*clpP*	**F61**	Full (aa 1–203)	25.34	< 0:001	0.49	104.75	**I203**
	**F1X4**	Full (aa 1–203)	26.96	< 0:001	0.49	83.13	**I203**
	**F3X4**	Full (aa 1–203)	27.41	< 0:001	0.5	114.9	**I203**
*ycf2*	**F61**	Full (aa 1–2183)	5.84	0:054	1.59	11.29	D65; R1147; K1190; N1238; K1571; H1655; L2048; A215
	**F1X4**	Full (aa 1–2183)	6.62	0:036	3.06	8.87	D65; R1147; K1190; N1238; K1571; H1655; L2048; A2155
	**F3X4**	Full (aa 1–2183)	6.83	0:033	4.67	7.27	D65; R1147; K1190; N1238; K1571; H1655; L2048; A2155

*P*-values were achieved by performing chi-squared tests on twice the difference of the computed log likelihood values of the models disallowing (M7) or allowing (M8) dN = dS > 1. The BEB column lists rapidly evolving sites with a dN = dS > 1 and a posterior probability > 0:95, determined by the Bayes Empirical Bayes implemented in Codeml. Amino acids refer to *C*. *istanbulensis* cp exonic sequence. Note that INDELs and the stop codon were removed from the alignment prior to evolutionary analysis, so shown positions are based on the alignment without gaps (aa = amino acids, *PP* = posterior probability).

### Estimating sequence distances between *C*. *istanbulensis* specimens

**Fig 5:**
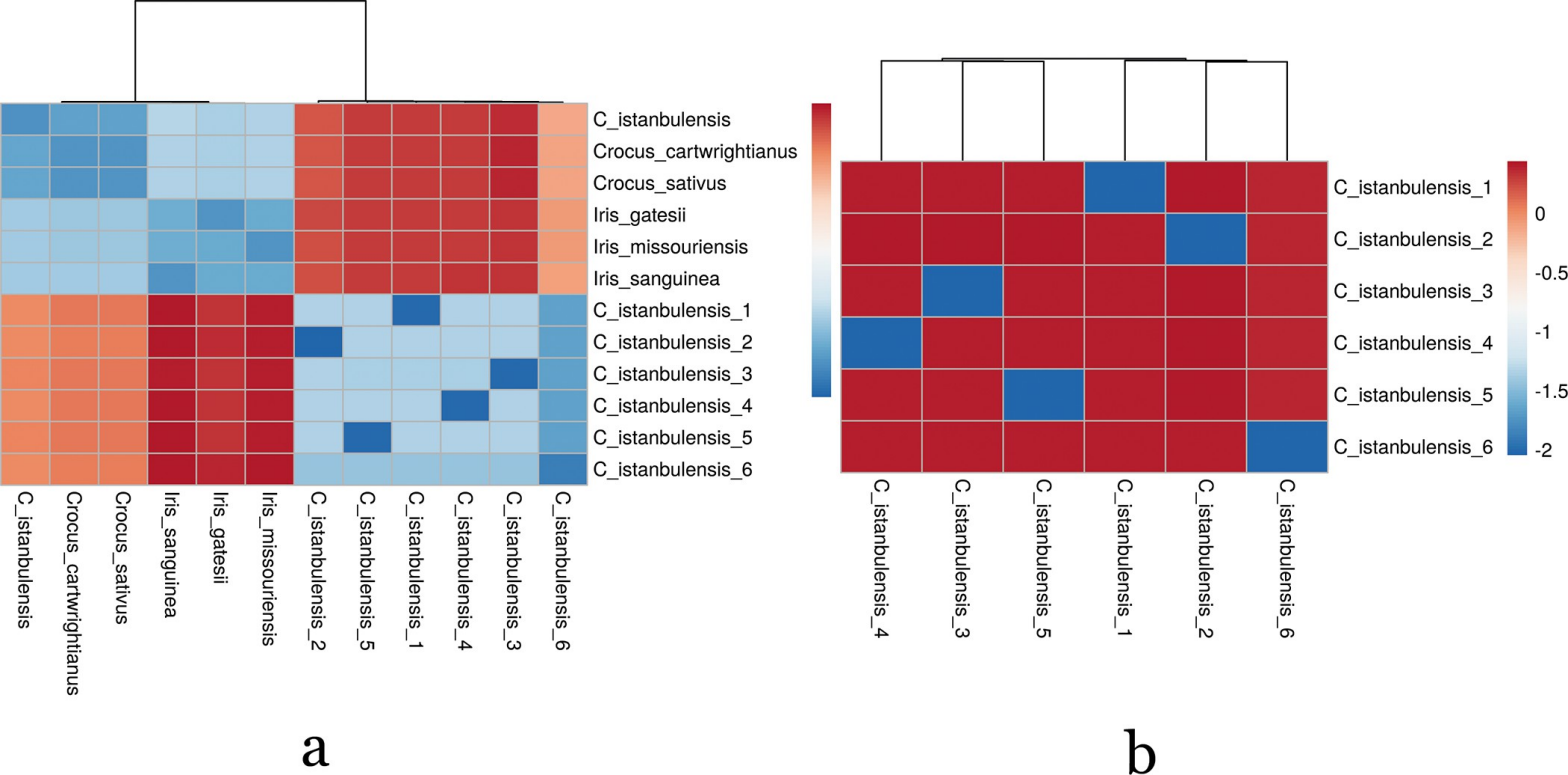
(a) A schematic representation of k-mer based unassembled similar results of *C*. *istanbulensis* individuals using genome skim data. (b) The sequence similarity results of *C*. *istanbulensis* individuals with the whole chloroplast genomes of other Iridaceae species. Warm colors (red) represent relatively moderate sequence diversity, whereas cool colors (blue) represent low sequence diversity.

We used Skmer [24] software to infer evolutionary distances between DNA sequences by calculating dissimilarity high-throughput sequencing reads of *C*. *istanbulensis*. Skmer, a relatively new approach, uses the minhash Jaccard similarity between sets of *k*-mers in sequences to estimate average nucleotide divergence among samples. Skmer-like approaches are preferred in genome skimming studies [84–86] because they can be applied to unassembled or assembled reads and deal with low sequencing coverage. We processed unassembled fastq files of *C*. *istanbulensis* as input assembly-free sequence distance estimates from low coverage genome skimming using Skmer. After generating a reference library and computing all pairwise distances, we queried the unassembled reads of *C*. *istanbulensis* against the reference library, producing a list of samples sorted by their distance to the query. The DNA sequence similarity among individuals from *C*. *istanbulensis* was found to be high based on *k*-mer analysis of genome skims (Fig 5).

Fig 5 shows homogeneous the distribution of sequence similarities among *C*. *istanbulensis*, indicating that the average nucleotide diversity is low, as expected (Fig 5A). We compared the unassembled reads of all *C*. *istanbulensis* individuals with the whole chloroplast genomes of other Iridaceae species (*C*. *istanbulensis*, *C*. *cartwrightianus*, *C*. *sativus*, *I*. *missouriensis*, *I*. *sanguinea*, *I*. *gatesii* and *G*. *australiensis*) using same the approach. As expected, there is a relatively high sequence diversity among Iridaceae species, while a low sequence diversity was noted among genome skim data in *C*. *istanbulensis* individuals (Fig 5B). *Crocus* species can reproduce by seed as well as vegetatively, spreading rapidly by forming small cormlets, or stolons as in *C*. *thirkeanus* K.Koch. and *C*. *kotschyanus* K.Koch. Vegetative reproduction usually takes place when the plant is under physiological stress. Stressors such as unfavorable corm depth, injury, and insufficient drainage may trigger cormlet reproduction. There have been few studies on the vegetative propagation of wild *Crocus* species [87–89]. This type of reproduction, which allows the plant to multiply rapidly, ensuring the reproduction and survival of the plant under stress, has a negative effect on genetic diversity. The low nucleotide diversity in the examined individuals may suggests vegetative reproduction in *C*. *istanbulensis*.

## Conclusions

We characterize the complete chloroplast genome sequence of six *C*. *istanbulensis* individuals, which is considered among the most endangered *Crocus* species in the world. We *de novo* assembled chloroplast genomes using genome skimming sequencing and focused on comparative analyses with other Iridaceae taxa. In general, the *C*. *istanbulensis* cp genome exhibited a pattern similar to other Iridaceae in terms of genome length, gene content and typical quadripartite structure. However, one inversion in the terminal positions of the LSC region and three different gene (*psbA*, *rps3* and *rpl22*) arrangements that have not been reported previously in Iridaceae were found in *C*. *istanbulensis*. To the best of our knowledge, this is the first work to detect a total of seven genes (*accD*, *rpoC2*, *psbK*, *rps12*, *ccsA*, *clpP* and *ycf2*) under positive selection in *Crocus* cp genomes. *C*. *istanbulensis* is currently known from only one population; however, should new populations be discovered, these findings will serve as comparison material and inform conservation studies. In summary, our results might contribute to further research on population genetics studies, help in conservation efforts for this threatened species and, shed light on the evolutionary history of *C*. *istanbulensis*.

## Supporting information

S1 Fig Schematic representation of chromosomal inversion breakpoint location in *C*. *istanbulensis* cp genome. (TIF)

S1 Table BGI-Seq 500 DNA nanoball sequencing and chloroplast genome mapping statistics. All six sequences were produced in this study (SRA accession numbers SRX7512825- SRX7512830).
(DOCX)

S2 Table Distribution and number of the protein-coding, transfer RNA and ribosomal RNA genes in seven Iridaceae species. (DOCX)

S3 Table List of repeated sequences in the chloroplast genomes of Iridaceae [IGS: intergenic sequence, * overlapped repeat region] (A: *Crocus istanbulensis*, B: *Crocus cartwrightianus*, C: *Crocus sativus*, D: *Iris sanguinea*, E: *Iris missouriensis*, F: *Iris gatesii*, G: *Geosiris australiensis*). (DOCX)
