# Supplementary figures and images for: Genome skimming approach reveals the gene arrangements in the chloroplast genomes of the highly endangered Crocus L. species: Crocus istanbulensis (B.Mathew) Rukšāns
Source: PLoS One. 2022 Jun 15;17(6):e0269747. doi: 10.1371/journal.pone.0269747 (PMC9200356; doi:10.1371/journal.pone.0269747)

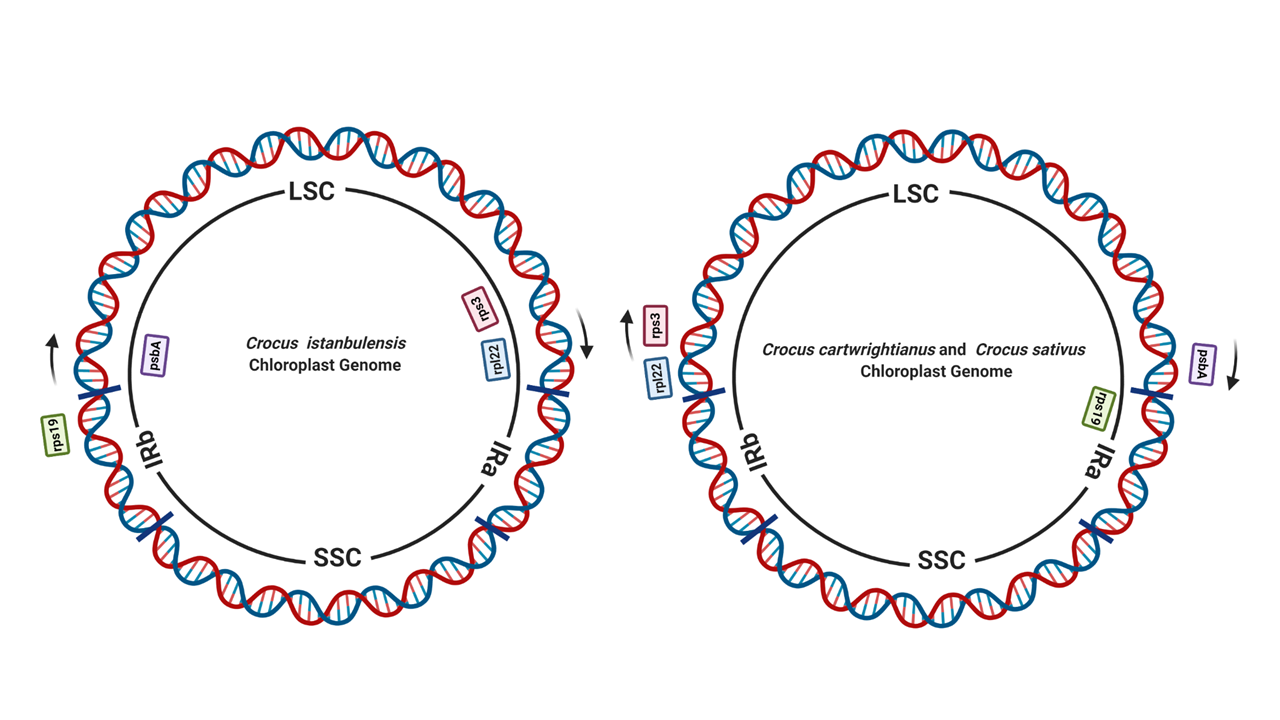

Supplement: S1 Fig — (TIF) [file pone.0269747.s001.tif]
