# Supplementary material for: Genome skimming approach reveals the gene arrangements in the chloroplast genomes of the highly endangered Crocus L. species: Crocus istanbulensis (B.Mathew) Rukšāns
Source: PLoS One. 2022 Jun 15;17(6):e0269747. doi: 10.1371/journal.pone.0269747 (PMC9200356; doi:10.1371/journal.pone.0269747)
Supplement: S1 Table — All six sequences were produced in this study (SRA accession numbers SRX7512825- SRX7512830). (DOCX) [file pone.0269747.s002.docx]

**S1 Table.** BGI-Seq 500 DNA nanoball sequening and chloroplast genome mapping statistics. All six sequences were produced in this study (SRA accession numbers SRX7512825- SRX7512830).

| SRR Acc. Num. | File | Format | Type | Num_seqs | Sum_len (bp) | Read_len (nt) | Q20 | Q30 | QC-passed mapped reads (PE100) | Average depth (X) |
| --- | --- | --- | --- | --- | --- | --- | --- | --- | --- | --- |
| SRX7512825 | V300017790_L3_505_1.fq.gz | FASTQ | DNA | 8,679,823 | 867,982,300 | 100 | 96.29 | 86.29 | 732378  ~73.2 million bp | 484 |
|  | V300017790_L3_505_2.fq.gz | FASTQ | DNA | 8,679,823 | 867,982,300 | 100 | 94.59 | 83.17 |  |  |
| SRX7512826 | V300017790_L3_506_1.fq.gz | FASTQ | DNA | 9,898,653 | 989,865,300 | 100 | 96.29 | 86.33 | 838419  ~83.8 million bp | 554 |
|  | V300017790_L3_506_2.fq.gz | FASTQ | DNA | 9,898,653 | 989,865,300 | 100 | 94.5 | 82.98 |  |  |
| SRX7512827 | V300017790_L3_507_1.fq.gz | FASTQ | DNA | 9,916,567 | 991,656,700 | 100 | 96.33 | 86.42 | 830889  ~83.0 million bp | 549 |
|  | V300017790_L3_507_2.fq.gz | FASTQ | DNA | 9,916,567 | 991,656,700 | 100 | 94.55 | 82.97 |  |  |
| SRX7512828 | V300017790_L3_508_1.fq.gz | FASTQ | DNA | 11,149,329 | 1,114,932,900 | 100 | 96.23 | 86.24 | 941422  ~94.1 million bp | 622 |
|  | V300017790_L3_508_2.fq.gz | FASTQ | DNA | 11,149,329 | 1,114,932,900 | 100 | 94.82 | 83.78 |  |  |
| SRX7512829 | V300019193_L4_505_1.fq.gz | FASTQ | DNA | 7,467,670 | 746,767,000 | 100 | 98.23 | 92.86 | 638776  ~63.8 million bp | 422 |
|  | V300019193_L4_505_2.fq.gz | FASTQ | DNA | 7,467,670 | 746,767,000 | 100 | 93.21 | 83.02 |  |  |
| SRX7512830 | V300019193_L4_506_1.fq.gz | FASTQ | DNA | 9,954,621 | 995,462,100 | 100 | 98.11 | 92.48 | 852079  ~85.2 million bp | 563 |
|  | V300019193_L4_506_2.fq.gz | FASTQ | DNA | 9,954,621 | 995,462,100 | 100 | 92.4 | 81.69 |  |  |
| *Total Read and Bases* | | | | **114,133,326** | **11,413,332,600** |  | | | ***Average Depth*** | 532X |
