# Supplementary material for: Genome skimming approach reveals the gene arrangements in the chloroplast genomes of the highly endangered Crocus L. species: Crocus istanbulensis (B.Mathew) Rukšāns
Source: PLoS One. 2022 Jun 15;17(6):e0269747. doi: 10.1371/journal.pone.0269747 (PMC9200356; doi:10.1371/journal.pone.0269747)
Supplement: S2 Table — (DOCX) [file pone.0269747.s003.docx]

**S2 Table.** Distribution and number of the protein-coding, transfer RNA and ribosomal RNA genes in seven Iridaceae species.

| Region | Gene | *Crocus istanbulensis* | *Crocus cartwrightianus* | *Crocus*  *sativus* | *Iris*  *sanguinea* | *Iris*  *gatesii* | *Iris missouriensis* | *Geosiris australiensis* |
| --- | --- | --- | --- | --- | --- | --- | --- | --- |
| LSC | PGGs | 62 | 62 | 62 | 64 | 62 | 62 | 21 |
|  | tRNAs | 21 | 21 | 21 | 21 | 21 | 21 | 20 |
|  | rRNAs | - | - | - | - | - | - | - |
| IRb | PGGs | 6 | 6 | 6 | 6 | 6 | 6 | 9 |
|  | tRNAs | 8 | 8 | 8 | 8 | 8 | 8 | 8 |
|  | rRNAs | 4 | 4 | 4 | 4 | 4 | 4 | 4 |
| SSC | PGGs | 12 | 12 | 12 | 11 | 12 | 12 | - |
|  | tRNAs | 1 | 1 | 1 | 1 | 1 | 1 | 1 |
|  | rRNAs | - | - | - | - | - | - | - |
| IRa | PGGs | 6 | 6 | 6 | 6 | 6 | 6 | 9 |
|  | tRNAs | 8 | 8 | 8 | 8 | 8 | 8 | 8 |
|  | rRNAs | 4 | 4 | 4 | 4 | 4 | 4 | 4 |
