# Supplementary material for: Genome skimming approach reveals the gene arrangements in the chloroplast genomes of the highly endangered Crocus L. species: Crocus istanbulensis (B.Mathew) Rukšāns
Source: PLoS One. 2022 Jun 15;17(6):e0269747. doi: 10.1371/journal.pone.0269747 (PMC9200356; doi:10.1371/journal.pone.0269747)
Supplement: S3 Table — (DOCX) [file pone.0269747.s004.docx]

**S3 Table.** List of repeated sequences in the chloroplast genomes of Iridaceae [IGS: intergenic sequence, * overlapped repeat region] (**A:***Crocus istanbulensis*, **B:***Crocus cartwrightianus*, **C:***Crocus sativus*, **D:***Iris sanguinea*, **E:***Iris missouriensis*, **F:***Iris gatesii*, **G:***Geosiris australiensis*).

| Repeat lenght (bp) | Start site of repeat A | Repeat A location | Repeat A region | Start site of repeat B | Repeat B location | Repeat B region | Repeat Type |
| --- | --- | --- | --- | --- | --- | --- | --- |
| 52 | 53595 | IGS ( *psbM* - *petN*) | LSC | 53595 | IGS( *psbM* - *petN*) | LSC | P |
| 32 | 74028 | IGS ( *trnS* - *psbI*) | LSC | 74028 | IGS *(trnS* - *psbI*) | LSC | P |
| 31 | 124486 | *ycf1* | SSC | 124498 | *ycf1* | SSC | F |
| 30 | 81135 | IGS ( *psbA* - *rps19*) | LSC | 81168 | IGS ( *psbA* - *rps19*) | LSC | P |
| 30 | 110000 | IGS ( *ndhF* - *trnL*) | IR | 110000 | IGS ( *psbA* - *rps19*) | IR | P |

**(A)** *Crocus istanbulensis*

**(B)** *Crocus cartwrightianus*

| Repeat lenght (bp) | Start site of repeat A | Repeat A location | Repeat A region | Start site of repeat B | Repeat B location | Repeat B region | Repeat Type |
| --- | --- | --- | --- | --- | --- | --- | --- |
| 56 | 54137 | IGS (*rbcL* - *accD*) | LSC | 54137 | IGS (*rbcL* - *accD*) | LSC | P |
| 53 | 124220* | *ycf1* | SSC | 124232 | *ycf1* | SSC | F |
| 52 | 27435 | IGS (*petN* - *psbM*) | LSC | 27435 | IGS (*petN* - *psbM*) | LSC | P |
| 41 | 124220* | *ycf1* | SSC | 124244 | *ycf1* | SSC | F |
| 32 | 114501 | IGS (*psaC* - *ndhE*) | SSC | 114501 | IGS (*psaC* - *ndhE*) | SSC | P |
| 31 | 55158 | *accD* | LSC | 55164 | *accD* | LSC | F |

**(C)** *Crocus sativus*

| Repeat lenght (bp) | Start site of repeat A | Repeat A location | Repeat A region | Start site of repeat B | Repeat B location | Repeat B region | Repeat Type |
| --- | --- | --- | --- | --- | --- | --- | --- |
| 56 | 54137 | IGS (*rbcL* - *aacD*) | LSC | 54137 | IGS (*rbcL* - *aacD*) | LSC | P |
| 53 | 124221* | *ycf1* | SSC | 124233 | *ycf1* | SSC | F |
| 52 | 27435 | IGS (*petN* - *psbM*) | LSC | 27435 | IGS (*petN* - *psbM*) | LSC | P |
| 41 | 124221* | *ycf1* | SSC | 124245 | *ycf1* | SSC | F |
| 32 | 114502 | IGS (*psaC* - *ndhE*) | SSC | 114502 | IGS (*psaC* - *ndhE*) | SSC | P |
| 31 | 55158 | *accD* | LSC | 55164 | *accD* | LSC | F |

**(D)** *Iris sanguinea*

| Repeat lenght (bp) | Start site of repeat A | Repeat A location | Repeat A region | Start site of repeat B | Repeat B location | Repeat B region | Repeat Type |
| --- | --- | --- | --- | --- | --- | --- | --- |
| 54 | 28796 | *PetN* | LSC | 28796 | *PetN* | LSC | P |
| 48 | 115135 | IGS (*ndhD* - *psaC*) | SSC | 115135 | IGS (*ndhD* - *psaC*) | SSC | P |
| 42 | 87600* | *ycf2* | IR | 87621 | *ycf2* | IRb | F |
| 42 | 87600* | *ycf2* | IR | 147085 | *ycf2* | IRa | P |
| 42 | 87621* | *ycf2* | IR | 147106 | *ycf2* | IRa | P |
| 42 | 147085 | *ycf2* | IR | 147106 | *ycf2* | IRa | F |

**(E)** *Iris missouriensis*

| Repeat lenght (bp) | Start site of repeat A | Repeat A location | Repeat A region | Start site of repeat B | Repeat B location | Repeat B region | Repeat Type |
| --- | --- | --- | --- | --- | --- | --- | --- |
| 52 | 28835 | IGS (*petN* - *psbM*) | LSC | 28835 | IGS (*petN* - *psbM*) | LSC | P |
| 48 | 119792 | IGS (*psaC* - *ndhD*) | SSC | 119792 | IGS (*psaC* - *ndhD*) | SSC | P |
| 36 | 5916 | IGS (*rps16* - *trnQ-UUG*) | LSC | 5916 | IGS (*rps16* - *trnQ-UUG*) | LSC | P |
| 31 | 87703* | *ycf2* | IR | 87724 | *ycf2* | IR | F |
| 31 | 87703* | *ycf2* | IR | 147726 | *ycf2* | IR | P |
| 31 | 87724* | *ycf2* | IR | 147747 | *ycf2* | IR | P |
| 31 | 109600 | *ycf1* | SSC | 109600 | *ycf1* | SSC | R |
| 31 | 147726 | *ycf2* | IR | 147747 | *ycf2* | IR | F |
| 30 | 109602 | *ycf1* | SSC | 109602 | *ycf1* | SSC | R |

**(F**) *Iris gatesii*

| Repeat lenght (bp) | Start site of repeat A | Repeat A location | Repeat A region | Start site of repeat B | Repeat B location | Repeat B region | Repeat Type |
| --- | --- | --- | --- | --- | --- | --- | --- |
| 94 | 0 |  | LSC | 82702 | IGS (*rpl22* - *rps19*) | IRb | P |
| 94 | 0 |  | LSC | 153347 | *rps19* | IRa | F |
| 52 | 5977 | IGS (*rps16* - *trnQ-UUG*) | LSC | 5977 | IGS (*rps16* - *trnQ-UUG*) | LSC | P |
| 52 | 29183 | IGS (*petN* - *psbM*) | LSC | 29183 | IGS (*petN* - *psbM*) | LSC | P |
| 48 | 115940 | IGS (*ndhD* - *psaC*) | SSC | 115940 | IGS (*ndhD* - *psaC*) | SSC | P |
| 42 | 87964* | *ycf2* | IR | 87985 | *ycf2* | IR | F |
| 42 | 87964* | *ycf2* | IR | 148116 | *ycf2* | IR | P |
| 42 | 87985 | *ycf2* | IR | 148137 | *ycf2* | IR | P |
| 42 | 148116 | *ycf2* | IR | 148137 | *ycf2* | IR | F |

**(G**) *Geosiris australiensis*

| Repeat lenght (bp) | Start site of repeat A | Repeat A location | Repeat A region | Start site of repeat B | Repeat B location | Repeat B region | Repeat Type |
| --- | --- | --- | --- | --- | --- | --- | --- |
| 48 | 46572 | IGS( *rps3* - *rpl22*) | IRb | 118169 | *rpl22-2* | IRa | F |
| 34 | 77000* | *ycf1* | IRb | 77001 | *ycf1* | IRb | F |
| 34 | 87754* | *ycf1-2* | IRa | 87755 | *ycf1-2* | IRa | F |
| 33 | 77000* | *ycf1* | IRb | 77002 | *ycf1* | IRb | F |
| 33 | 87754* | *ycf1-2* | IRa | 87756 | *ycf1-2* | IRa | F |
| 32 | 49986 | *ycf2* | IRb | 50001 | *ycf2* | IRb | F |
| 32 | 52295 | *ycf2* | IRb | 52340 | *ycf2* | IRb | F |
| 32 | 77000* | *ycf1* | IRb | 77003 | *ycf1* | IRb | F |
| 32 | 87754* | *ycf1-2* | IRa | 87757 | *ycf1-2* | IRa | F |
| 32 | 112417 | *ycf2-2* | IRa | 112462 | *ycf2-2* | IRa | F |
| 32 | 114756 | *ycf2-2* | IRa | 114771 | *ycf2-2* | IRa | F |
| 31 | 77000* | *ycf1* | IRb | 77004 | *ycf1* | IRb | F |
| 31 | 87754* | *ycf1-2* | IRa | 87758 | *ycf1-2* | IRa | F |
| 30 | 77000* | *ycf1* | IRb | 77005 | *ycf1* | IRb | F |
| 30 | 87754* | *ycf1-2* | IRa | 87759 | *ycf1-2* | IRa | F |
